# Implementing Telemedicine for Neonatal Care: Tutorial on a Practical Toolkit Based on Multinational Experience

**DOI:** 10.2196/90592

**Published:** 2026-09-30

**Authors:** Tereza Schedler, Brechtje Krijvenaar, Robyn Dvorsky, H Rob Taal, Irwin K M Reiss, Michael Wagner, Saba Hinrichs-Krapels, Jennifer Fang, Josephine H L Wagenaar

**Affiliations:** 1 Divison of Neonatology, Pediatric Intensive Care and Neuropediatrics, Comprehensive Center for Pediatrics, Department of Pediatrics and Adolescent Medicine, Medical University of Vienna, Vienna, Vienna, Austria; 2 Department of Design Organization and Strategy, Faculty of Industrial Design Engineering, Delft University of Technology, Delft, South Holland, The Netherlands; 3 Department of Neonatal and Paediatric Intensive Care, Erasmus MC - Sophia Children’s Hospital, Dr. Molewaterplein 40, Rotterdam, South Holland, 3015GD, The Netherlands, 31 107040704; 4 Division of Neonatology and Pediatric Intensive Care Medicine, University Medical Center Hamburg-Eppendorf, Hamburg, Hamburg, Germany; 5 Health Institute, Faculty of Technology Policy and Management, Delft University of Technology, Delft, South Holland, The Netherlands; 6 Division of Neonatal Medicine, Mayo Clinic College of Medicine and Science, Mayo Clinic, Rochester, MN, United States

**Keywords:** newborn, telemedicine, implementation, neonatal intensive care, organization and administration

## Abstract

Real-time audiovisual connections between health care providers (HCPs) in neonatal care, known as TeleNeonatology (TeleNeo), can improve neonatal care. For patients and families, TeleNeo was found to improve patient outcomes and facilitate family-integrated and patient-centered care by ensuring timely access to expert involvement regardless of location, which can strengthen trust and reassurance. For clinicians and health care organizations, TeleNeo enables expert decision-making, fosters continuous professional development, promotes knowledge exchange between hospitals, and increases staff confidence in managing complex medical cases. However, organizational, technical, and infrastructural requirements can hinder successful implementation and sustained adoption of technological interventions, such as TeleNeo. Implementation can be time-consuming and may fail due to the challenges encountered during the implementation process, particularly when incorporating the intervention into existing workflows across multiple hospitals. Nevertheless, there are case studies that demonstrate successful implementation of TeleNeo into routine care. Informed by international experience and theoretical underpinnings of implementation science, this tutorial presents a toolkit to provide step-by-step guidance for health care institutions considering TeleNeo implementation. The objective of the toolkit is to help health care organizations effectively and efficiently implement TeleNeo in their neonatal care pathways. The toolkit provides a structured guide that encompasses the entire implementation process, from initial ideas to stakeholder engagement, workflow design, and program evaluation. It includes checklists, planning guidelines, and tools to help teams design a customized implementation strategy for their institution.

## Introduction

TeleNeonatology (TeleNeo) is the real-time audiovisual connection between health care providers (HCPs) in neonatal care. The primary objective of this technology is to enhance the quality of care by connecting remote experts with local HCPs. TeleNeo can improve the quality of care by enhancing clinical decision-making and reducing transfers between hospitals [1-4]. TeleNeo has also been shown to benefit HCPs (neonatologists, neonatal nurses, nurse practitioners, and other members of the care team, as well as clinicians from other local or community hospitals) and the parents of neonatal patients [5]. In addition to its clinical application, telemedicine can be used as a tool for simulation-based training. Conducting simulation training via telemedicine enables learners to access remote expertise while developing knowledge and skills in both the use of telemedicine technologies and neonatal resuscitation [6].

However, implementing technology-based programs such as TeleNeo is challenging. Barriers may include limited resources, integrating telemedicine into existing workflows, and staff perceptions or attitudes toward telemedicine [2,4]. Furthermore, implementation requires time and active effort, a key point that is often overlooked and can be challenging, as most clinicians have a high patient-care workload [7]. As a result, ineffective implementation and nonadoption of technological innovations are well-known challenges throughout health care [8].

Multiple frameworks, such as the World Health Organization (WHO) telemedicine implementation guidelines [9], have been developed to assist clinicians with the implementation process of telemedicine. Furthermore, structured approaches have been published to explore barriers for implementation [10]. Although these tools have the potential to facilitate successful implementation, they are general guides intended to be applicable for various technological innovations. As such, they are not use case-specific and do not cover the broad range of implementation aspects needed for sustainable adoption.

Because implementation extends beyond simply installing the technology, we defined “successful implementation” as the sustained adoption and embedding of a technology within a health care organization, its care delivery processes, and its technical and organizational environment. This definition is commonly used in broader implementation science frameworks [11]. To achieve sustainable adoption and embedding within care processes, it is important to follow a structured approach that engages all relevant stakeholders from the beginning [11,12]. Furthermore, using clear visuals and consistent communication can help ensure smooth implementation and long-term success.

To facilitate this process and contribute to the successful implementation of telemedicine in neonatal care, we developed an easy-to-use, step-by-step TeleNeo implementation toolkit, informed by over 15 years of combined TeleNeo experience in the United States and Europe within different health care systems.

## Experience of the Research Team

Based on the combined experience of 3 international TeleNeo implementation teams (Mayo Clinic, Medical University of Vienna, and Erasmus Medical Center [MC]), ranging from large-scale clinical programs to simulation-based training and regional scaling programs, this toolkit builds on practical, real-world insights gained across diverse neonatal care settings.

Mayo Clinic’s TeleNeo program was established in 2013, supports 20 community hospitals across 3 states, and has provided over 1200 consultations, primarily focused on newborn resuscitation and stabilization [13]. The Mayo Clinic is also the sponsor and coordinating site for the TELENEO Trial, a North American multicenter clinical trial evaluating the impact of TeleNeo on the early health outcomes of at-risk newborns delivered in community hospitals (NCT06087224) [14,15]. At the Medical University of Vienna, TeleNeo has been implemented in the Department of Neonatology for training purposes within a simulated environment, with a primary focus on newborn life support scenarios. Currently, efforts are underway to implement TeleNeo for clinical use across Austria, as well as to further expand its application in simulation training. Erasmus MC implemented a TeleNeo program in 2 hospitals in the Netherlands for both acute consultations prior to potential transfer to a higher-level neonatal intensive care unit (NICU) and elective consultations surrounding back transfers to a lower-level NICU. This program aimed to reduce neonatal transfers and improve the quality of care, parental experience, and job satisfaction among HCPs [4]. At the time of writing this paper, preparations are being made to scale the TeleNeo program to 8 other hospitals in the region.

## Derivation of the Toolkit

### TeleNeo Toolkit Development Process

The TeleNeo toolkit was developed using an iterative design process, informed by underlying literature and guidance (Figure 1), real-life experience with telemedicine programs, as well as repeated cycles of reflection and refinement across multiple health care systems. This approach has been shown to strengthen the feasibility and sustainability of complex eHealth implementations [16]. The development process combined experiential knowledge, structured reflection, literature review, and stakeholder feedback to create a usable implementation guide for HCPs and staff in neonatal departments. The toolkit was created by an interdisciplinary, international team with experience in neonatal health care, telemedicine, quality improvement, program design, and research. This diverse composition enabled a comprehensive view of the implementation process from various perspectives.

The methodology consists of 5 phases, starting with “mapping existing TeleNeo practices” and ending with “final refinement of the toolkit.” These phases are visualized in Figure 1 and explained in more detail below.

**Figure 1.**
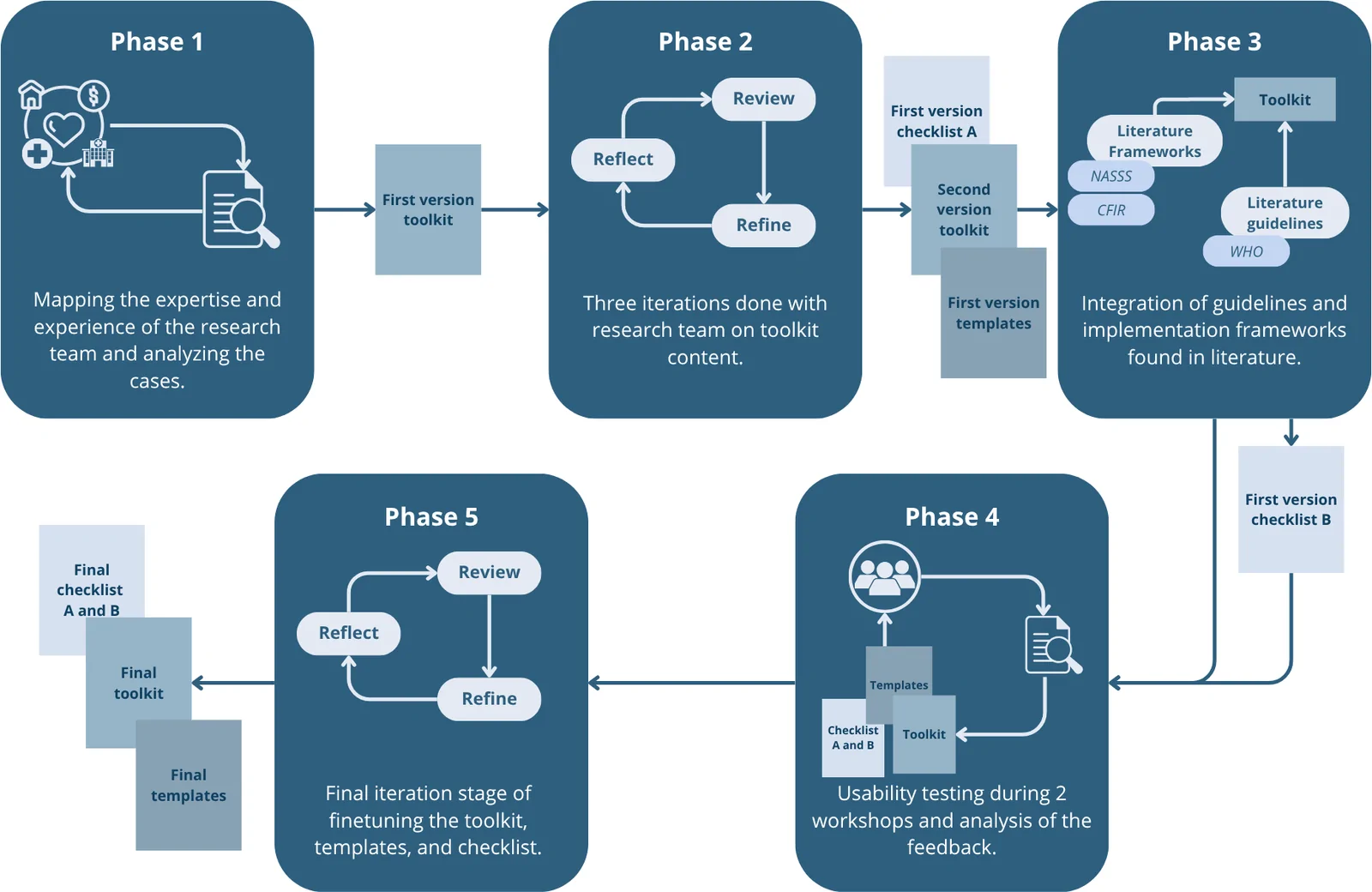
Iterative methodology for the development of the TeleNeo toolkit, templates, and checklists. CFIR: Consolidated Framework for Implementation Research; NASSS: nonadoption, abandonment, scale-up, spread and sustainability; WHO: World Health Organization.

### Phase 1: Mapping Existing TeleNeo Practices

The first phase focused on mapping and analyzing existing TeleNeo implementation practices at 3 institutions: the Mayo Clinic in Rochester, Minnesota (United States), the Medical University of Vienna (Austria), and the Erasmus MC in Rotterdam (the Netherlands). Together, these institutions represent a combined TeleNeo experience across diverse health care systems and use cases, including acute clinical teleconsultations, elective consultations, and tele-simulation–based education.

During phase 1, key implementation activities, decision points, and organizational requirements for each use case were outlined by team members (JF and JHLW). This resulted in an initial list of implementation steps capturing recurring activities across the 3 programs, including stakeholder engagement, workflow (re)design, technology selection, finances and budgeting, staff training, and monitoring and evaluation.

### Phase 2: Synthesis and Reflection

In the second phase, the focus shifted toward engaging in structured reflection sessions to review, refine, and discuss the initial list of implementation steps. Differences across the 3 institutional contexts were discussed to distinguish the core implementation elements and, therefore, the steps in the toolkit itself. This discussion helped to identify elements that could easily be adapted to specific contexts.

During this phase, early versions of the toolkit, templates, and checklists were created and iteratively revised. Decisions focused on clarity of steps, usability of the toolkit, and feasibility for clinical teams with limited time and resources. These iterations led to the simplification of the language used, the consolidation of overlapping steps, and the introduction of visual templates and checklists to support discussion among stakeholders. Two prior versions of the toolkit, as well as the templates generated during this phase, can be found in Multimedia Appendix 1, which illustrate the iterative process.

### Phase 3: Integration of Literature and Implementation Frameworks

To strengthen the conceptual basis of the toolkit, we referred to TeleNeo-specific literature (specifically, WHO telemedicine implementation guidance [9]) and established implementation science frameworks. Several frameworks used in implementation science provide valuable perspectives on implementation processes, aiming to guide the process of translating research into practice (process models), explain factors that influence implementation outcomes (determinant frameworks, classic theories, and implementation theories), and evaluate implementation efforts (evaluation frameworks). We referred to the NASSS (nonadoption, abandonment, scale-up, spread and sustainability) framework [10], the CFIR (Consolidated Framework for Implementation Research) [17], the RE-AIM (Reach, Effectiveness, Adoption, Implementation, and Maintenance) [12], and the MAST (Model for Assessment of Telemedicine) [18]. These frameworks were used as sensitizing tools to ensure that relevant implementation domains, such as organizational readiness, workflow integration, and evaluation, were considered during toolkit development. We also refer to these frameworks in our toolkit (see section 2: step 2.2) for users to consider the use of such a framework in their implementation approach.

Instead of embedding a single framework or requiring users to apply implementation theories, the key concepts from these frameworks were translated into practical questions, actions, and considerations within the steps of the toolkit. This approach ensured that the frameworks informed the structure and content of the toolkit while allowing users to apply additional frameworks as required in their own context.

### Phase 4: Usability Testing With Stakeholders

The preliminary version of the toolkit was evaluated through usability testing conducted during an interactive workshop held at the “Gesellschaft für Neonatologie und Pädiatrische Intensivmedizin” conference in Lübeck, Germany, in 2025. In total, 22 pediatricians worked through the toolkit sections in small groups using the templates (Multimedia Appendix 2). Feedback was collected through informal group discussions and individual comments. Divergent feedback was discussed within the research team and reconciled through iterative consensus, prioritizing changes that aligned with the intended logical sequence and usability of the toolkit. Participant feedback was positive, highlighting the usefulness of the toolkit to prompt discussions about the potential value of TeleNeo and to recognize the importance of involving multiple medical disciplines during the implementation process. Participants recommended simplifying terminology, clarifying the order of certain steps, and providing examples related to stakeholder involvement and financial considerations. Based on their feedback, the “potential approaches” column was added to the toolkit. Furthermore, participants tended to reason from specific technological modalities rather than starting from the underlying problem and requirements, which may constrain consideration of the full range of possible approaches. In response, the toolkit was revised to separate requirement specifications from technology selection, reinforcing a more structured, stepwise approach.

An additional workshop was held in the Netherlands, in February 2026, within the Erasmus MC region. The workshop involved 18 pediatricians from regional neonatal care networks. Feedback was collected through group discussion. The feedback was positive and mentioned that the visualization helped them to structure their thoughts and discuss the steps. The toolkit helped participants think more broadly about implementation before starting the process. Participants suggested that this would save them time during the next steps of implementation and potentially lead to a more effective implementation process due to earlier stakeholder involvement.

### Phase 5: Final Refinement of the Toolkit

Following the workshops, participant feedback was reviewed and discussed, and the relevant changes were incorporated into the toolkit. Adjustments to the toolkit included reordering steps, refining guiding questions, and improving consistency across different sections. Additionally, the visual templates were simplified to improve usability and feasibility within clinical settings. A final internal iteration among the research team ensured that the toolkit reflected shared priorities and lessons learned across institutions.

The resulting TeleNeo implementation toolkit is the final product of this process. Rather than being a formally validated intervention, it is intended as a practice-oriented guide to support health care organizations in planning and implementing TeleNeo programs. The toolkit is described in the following sections.

## TeleNeo Implementation Toolkit

### Overview and Structure of the TeleNeo Toolkit

The TeleNeo toolkit is a step-by-step guide to support the implementation of a neonatal telemedicine program. The toolkit can be applied to both clinical and educational settings. For instance, it can be used to implement tele-consultations and/or tele-simulation. Although telemedicine implementation in a simulation context differs from that in a clinical setting, the toolkit can still be applied to both. The most important factors for implementation in a simulated setting are a telecommunication platform, program design and debriefing, and aspects that are offered by the TeleNeo toolkit [6]. A multidisciplinary team, including HCPs, managers, and technicians, is recommended to ensure that all roles are included from the beginning.

The toolkit is organized into three sections: (1) developing a TeleNeo program, (2) preparing for implementation, and (3) program go-live and beyond. Each section includes practical resources such as checklists, planning guidelines, tools, and methods for evaluation. The toolkit sections and steps are summarized in Table 1, templates to guide discussion are presented in Figures 2 and 3, a stakeholder checklist in Multimedia Appendix 3, and suggested evaluation metrics in Multimedia Appendix 4.

**Table 1.** TeleNeonatology (TeleNeo) implementation toolkit.

Action	Considerations	Potential approaches
Section 1: developing a TeleNeo program (use template in Figure 2 to guide you through the steps)		
1.1 Identify the gap	What clinical, educational, or organizational challenges within the NICU^a^ could be addressed through telemedicine? To what extent could telemedicine help overcome limitations in network capabilities or resources?	Ask key users (parents, nurses, and physicians) for their thoughts on challenges and room for improvement. Search literature for evidence on TeleNeo impact.
1.2 Identify stakeholders—beneficiaries	Which stakeholders may benefit from implementing TeleNeo in care activities? What are the needs and preferences of potential beneficiaries? How can stakeholders be effectively involved and engaged, considering their availability, preferences, and accessibility?	Interview key users for insights in how to optimize the potential impact of TeleNeo from their perspective. Discuss first ideas with key stakeholders to create a shared idea of the innovation.
1.3 Identify stakeholders—partners and influentials	Which stakeholders are involved in delivering care via telemedicine? Which stakeholders are required to facilitate organizational and care pathway changes, and what is their level of influence? How can stakeholders be effectively involved and engaged, considering their availability, preferences, and accessibility?	Use stakeholder frameworks (eg, salience framework) to help determine the level of influence and dependence of specific stakeholders. See list of potential stakeholders in Multimedia Appendix 3. Consider the legal, financial, and technical aspects of organizational change.
Iteration**:** after talking with your stakeholders, go back to your gaps and use those answers to define the next steps below		
1.4 Specify TeleNeo activities	Based on the gaps found at 1.1; which care interactions and activities could be improved using telemedicine?	Consider which care activities could be supported by TeleNeo technology.
1.5 Define the telemedicine workflow	How do existing workflows change with the introduction of telemedicine? Which stakeholders are responsible for staffing the telemedicine service, monitoring its performance, and ensuring it meets the desired standards?	Involve key users (eg, nurses and physicians). Conduct site visits to gain an understanding of the impact of changes to the current workflow. Consider your organization’s unique documentation requirements.
1.6 Specify TeleNeo requirements	Which existing infrastructure, platforms, or protocols can support telemedicine? Do staff require additional licenses or hospital-specific credentials to provide telemedicine services?	Ask advice from specific experts (eg, policy) in your health system.
1.7 Technology selection	Which technical modalities are required to perform the telemedicine activities? Which requirements should telemedicine hardware and software meet in terms of functionality, performance, privacy, system integration, and data storage? Is a formal procurement process (eg, tender) required to support implementation?	Ask advice from specific experts (eg, technology) in your health system. Look for examples of telemedicine within and outside your own hospital.
1.8 Explore costs and finances	Which funding options are available to cover implementation costs (eg, research or innovation budgets)? What are the structural costs of the TeleNeo program (eg, licenses, technical requirements, and staffing)? What is the impact of the TeleNeo program on patient-related revenue, and which reimbursement structures are available?	Ask advice from financial controllers. Create a budget and preliminary business case.
Section 2: Preparing for implementation (use template in Figure 3 to guide you through the steps)		
2.1 Engage users	What is required to establish a shared vision and goals with key users?	Give presentations and use informal talks with key users.
2.2 Barriers for implementation	What potential physical and psychological barriers may arise during telemedicine implementation, and how can these be addressed? What measures are required to ensure clinical safety, data protection, and compliance with ethical standards during implementation?	Frequently used frameworks to explore barriers are NASSS^b^ [10] and CFIR^c^ [17]. Both include a toolkit to guide you through the framework. Ask advice from privacy, legal, and ethical experts.
2.3 Define timeline	When should key implementation milestones be completed to achieve the target go-live date? Which stakeholders should be involved at each stage of the timeline, and what resources are required?	Use the timeline on Figure 2 to define the steps.
2.4 Staff training program	How should training sessions be designed in terms of frequency, content, delivery method (professional or peer colleagues), and modality (eg, videos, in-person, or virtual reality; group or individual)? Which training materials are required to support follow-up questions and ongoing education? How can digital communication practices be integrated into training?	When possible, integrate with existing training sessions. Collaborate with educational experts. Include digital communication style into training.
2.5 Identify program evaluation metrics	Which process, outcome, cost, and experience measures should be evaluated? How can user feedback be collected and evaluated (eg, digital, anonymous, in-person, individually, or group-based)? How can the quality and performance of TeleNeo be monitored and continuously improved to ensure intended outcomes are achieved?	Search literature for methods used to evaluate TeleNeo program. For examples, explanations of evaluation methods for purposes Multimedia Appendix 4.
2.6 Legal and finances	Which legal contracts are required for the telemedicine program (eg, data transfer agreements, collaboration agreements, and liability arrangements)? Which stakeholders are responsible for approving the business case and financial impact of the TeleNeo program?	Involve legal and financial experts.
Section 3: program go-live and beyond (continue with template in Figure 3)		
3.1 Go live!	Which communication strategies and channels should be used to ensure that relevant staff and stakeholders are informed, engaged, and prepared for program launch?	Use existing communication channels, like newsletters, screensavers, or weekly meetings.
3.2 Program monitoring and continuous improvement	How can the performance of the TeleNeo program be tracked and visualized over time, considering the needs and preferences of users and stakeholders? How can TeleNeo usage be systematically, and where possible automatically, recorded to support continuous performance monitoring? Which stakeholders should be involved in updating the program and workflow, and how should their collaboration be organized (eg, designers, stakeholders, users, and initiators)?	Appoint a dedicated program manager. Schedule 30-day, 60-day, and/or 90-day post-go-live touchpoint meetings with key stakeholders to review program performance and identify opportunities for improvement. Ask technicians for advice on how to automatically track and visualize the TeleNeo program. Check with local IT companies if they have automatic registration services available. Collaborate and check with key stakeholders when updating the program.
3.3 Ongoing training	When and how should additional simulation training be offered, and which participant should be involved? How can new staff be introduced and prepared for the telemedicine program? Which resources, technologies, and scenario types are required to ensure meaningful and targeted simulation-based training?	Define a set of key training scenarios and create a standardized simulation script for each (including objectives, who is involved, and what equipment or setting is needed).

a NICU: neonatal intensive care unit.

b NASSS: nonadoption, abandonment, scale-up, spread and sustainability.

c CFIR: Consolidated Framework for Implementation Research.

**Figure 2.**
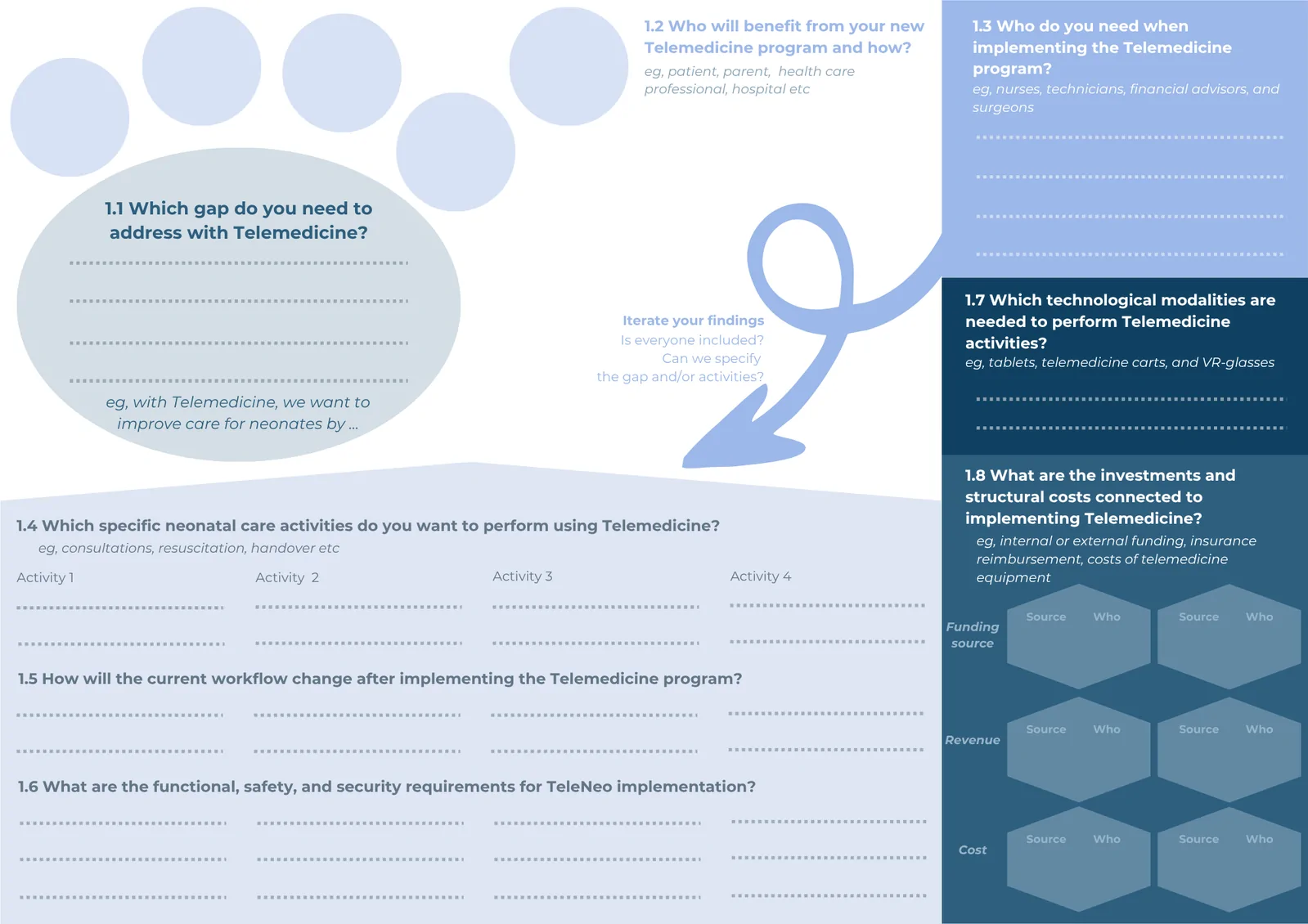
Develop your TeleNeo program. VR: virtual reality.

**Figure 3.**
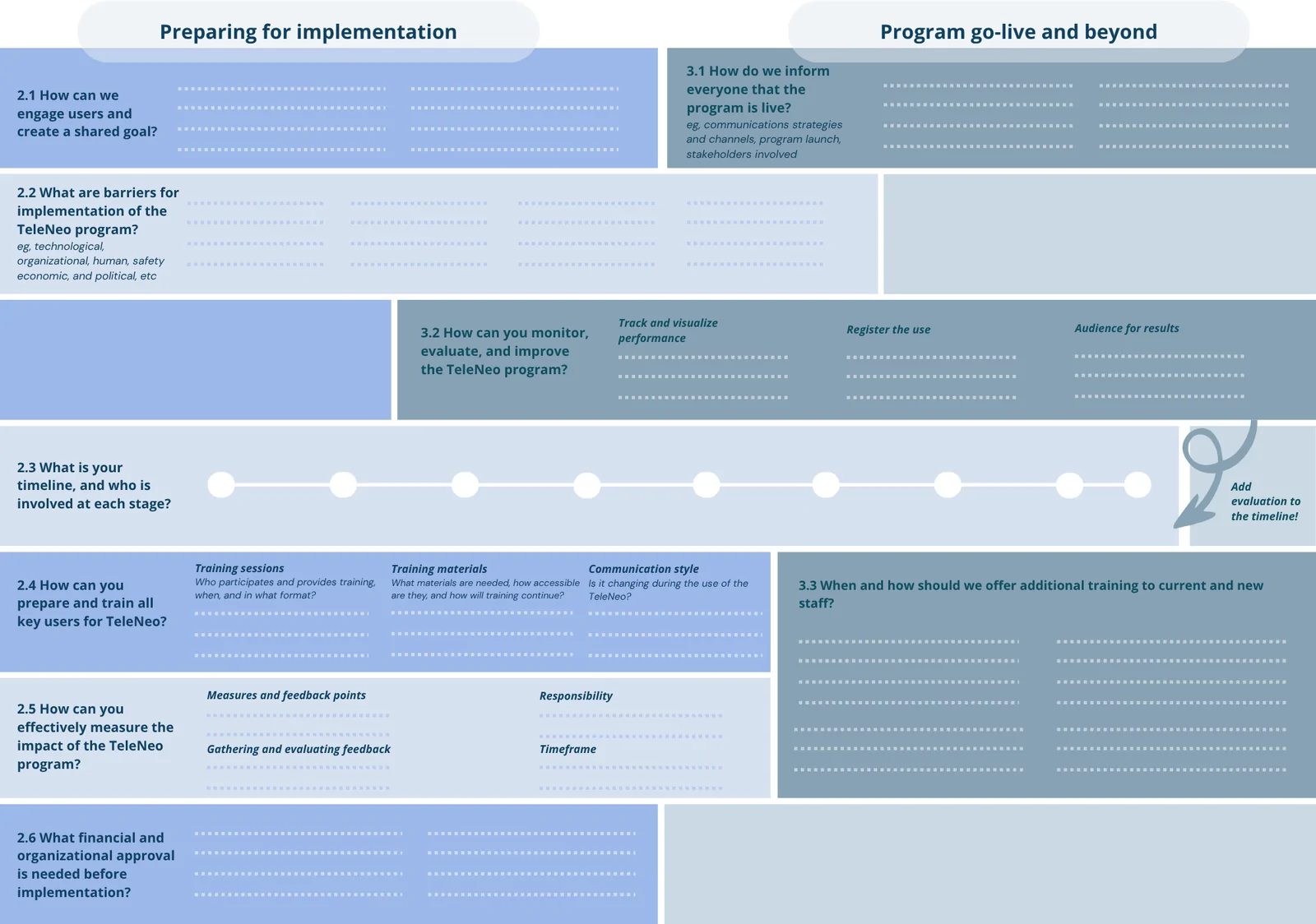
Prepare your implementation strategy and go live!

### Section 1: Developing a TeleNeo Program

Section 1 includes the foundational steps for developing a TeleNeo program: identifying the neonatal care gap that telemedicine can address, determining the program’s beneficiaries and their needs, and identifying the stakeholders required for implementation. Throughout this section, Figure 2, “develop your TeleNeo program” should be used.

The initial step in developing a TeleNeo Program is the systematic identification of gaps in neonatal care that can be addressed through telemedicine (step 1.1). This requires an assessment of existing clinical, educational, and organizational challenges within the NICU and referral region, as well as of the different disciplines involved.

After identifying the gap, it is essential to identify the primary beneficiaries of a TeleNeo program and determine how they may gain from its implementation (step 1.2). These could include patients and their families, as well as HCPs engaged in in-patient care, subspecialty consultations, and outpatient follow-up. Their needs, preferences, and preferred modes of engagement should be explored through interviews and discussions. Early involvement of these stakeholders helps to optimize the potential impact of the program and creates a shared understanding of its goals.

Successful implementation of TeleNeo requires the engagement of key partners and influential stakeholders (step 1.3). These include health care professionals, administration, operational/program management, legal and contracting, information technology teams, and financial staff. Their input and/or approval are necessary for adapting workflows, ensuring compliance, and facilitating structural integration. A structured identification process helps to ensure that all relevant actors are engaged at an early stage and that potential barriers to approval are minimized. To aid the team in identifying relevant stakeholders and key partners, a stakeholder checklist (Multimedia Appendix 3) can be used.

After step 1.3, an iteration can be conducted as needed based on the information and knowledge gathered regarding the clinical care gap(s) and stakeholders. This may better specify the neonatal care gap(s) from multiple perspectives and involvement of others in these activities. Iterating adds an additional deepening step to the implementation strategy.

With input from the identified stakeholders, TeleNeo activities can be further specified in step 1.4. This step translates the previously defined care gap(s) and stakeholder needs into concrete telemedicine use-cases that together form the TeleNeo program. When the implementation process begins with a clearly defined problem and use-case, this step represents a relatively straightforward operationalization of needs into specific TeleNeo activities, as illustrated by the Mayo Clinic experience, where the primary challenge was improving the quality of neonatal resuscitation in referring hospitals. In contrast, Erasmus MC experienced multiple challenges across the neonatal care pathway: potentially avoidable NICU transfers, suboptimal parental experiences and information exchange surrounding back transfers, and nurses’ shortages. Erasmus MC used step 1.4 to specify 3 TeleNeo activities: acute NICU consultations, warm handovers in collaboration with parents and both care teams, and joint daily rounds.

In collaboration with stakeholders, the project team could identify all contact moments between HCPs and/or parents and assess which interactions could be effectively supported by audiovisual guidance. Across organizations, this process may result in a range of TeleNeo activities, including prenatal consultations, remote resuscitation support, simulation-based education, support for NICU transfers, subspecialty consultations (eg, cardiology and neurology), virtual ward rounds, remote therapy, and multidisciplinary handovers to transfer hospitals or primary care providers [19,20].

Once activities have been selected, it is necessary to design a workflow that integrates TeleNeo into daily practice (step 1.5). Workflow development should include input from those who will be providing and receiving the telemedicine service, as well as any interrelated services, for example, medical transport and logistics. The new telemedicine workflow should be mapped from start to finish. This includes specifying the steps involved, defining who is responsible for each task, and clarifying documentation requirements. A detailed clinical service flow chart outlining the full process and associated roles and responsibilities should also be drafted to ensure clarity for training. Process maps or swim lane diagrams are useful tools for creating a comprehensive, organized TeleNeo workflow. The final workflow can be used to train all staff members, ensuring everyone is working from a shared understanding of the new process. The workflow can also be used to create quick reference guides that staff use in real-time during clinical care or simulation. When drafting the TeleNeo workflow, the project team should also address the staffing of the telemedicine service, integration with any interrelated existing clinical services, and opportunities to monitor service quality.

To guarantee safe and effective implementation, the technical, safety, privacy, and security requirements of the TeleNeo program must be clearly defined (step 1.6). This includes functionality, system performance, interoperability with existing platforms, data privacy, and secure storage of patient data (if needed). Institutions should evaluate whether existing telemedicine infrastructure can be leveraged or adapted. Expert input from technical specialists is critical for ensuring compliance with organizational standards and for selecting appropriate hardware and software solutions.

Selecting the appropriate technology (platform) is essential to ensure that telemedicine activities can be delivered reliably and securely (step 1.7 and the technology selection checklist in Multimedia Appendix 5). Depending on institutional requirements and budget specifications, platforms such as Zoom, Microsoft Teams, or WhatsApp (Meta Platforms, Inc) may be considered, each providing distinct levels of functionality, system integration, and data protection. Before using one of these platforms, institutions should always check for patient privacy and data security concerns. Alternatively, TeleNeo may use specialized TeleNeo carts equipped with high-resolution cameras controlled by the remote neonatologist, stationary units with permanently installed hardware, or augmented reality headsets to support real-time collaboration and clinical decision-making [21].

The final step in program design is the financial assessment of the TeleNeo program (step 1.8). This includes estimating initial investment costs, including telemedicine equipment and project management, as well as ongoing structural costs related to licenses, staffing, and technology maintenance. The impact of the program on reimbursements, program contractual payments, other patient-related income, as well as potential cost savings, must be integrated into the institutional business case. Developing a preliminary budget and business case in collaboration with engineering and technology maintenance staff, as well as financial experts and financial decision-makers, provides transparency and strengthens the basis for institutional decision-making.

### Section 2: Preparing for Implementation

Section 2 includes the key elements necessary for the successful implementation of TeleNeo, including engaging users, identifying barriers, defining the timeline, adapting workflows, developing training strategies, establishing evaluation methods, and defining finances. Throughout this section, Figure 3, “prepare your implementation strategy and go live!” should be used.

To create a shared future goal with key users, it is essential to engage them through presentations and informal discussions (step 2.1). Local champions should be selected to enhance engagement within local care teams. A local champion is a trusted clinician or staff member who actively promotes telemedicine adoption, serving as a role model whose engagement helps overcome resistance and facilitate successful implementation. Their visible engagement and advocacy are critical elements of change management, helping to overcome resistance and to embed new practices into routine care [22,23].

To properly prepare for implementation, it is important to identify the barriers that might hinder successful TeleNeo implementation (step 2.2). These barriers can be physical, such as infrastructural limitations, as well as mental, such as resistance to change by HCPs [4,7,15,24]. Identification and resolution of these issues can be facilitated by referencing Figure 3, “prepare your implementation strategy and go live!”. Implementation determinant frameworks such as NASSS and CFIR provide guidance on exploring implementation barriers. Both include useful step-by-step tools to identify these barriers [10,17] and are provided as supplementary tools. Supportive measures, including training, peer teaching, and support, can help reinforce commitment and knowledge of the local teams. Ensuring clinical safety, data protection, and compliance with ethical standards is essential. Implementation leaders should seek guidance from privacy, legal, and ethics committees that have valuable insights. In our experience, some of the most rate-limiting factors are state licensure (in the United States), hospital credentialing, security/privacy approvals, and contract execution.

To coordinate the process over time, a realistic timeline must be established. This involves defining milestones, allocating resources to specific phases, and identifying which stakeholders need to be involved at which points (step 2.3).

Furthermore, it is crucial to have a plan for staff training (step 2.4). Training can be offered in various formats, including in-person, digital, or virtual sessions, in either group or individual settings. When possible, this should be integrated into existing educational activities or existing workflows. Training sessions should include a review of the telemedicine workflow, equipment set-up and use, and any relevant communication and teamwork strategies. We also highly recommend that teams simulate the full TeleNeo workflow, for example, from service activation to the conclusion of the consult, prior to service go-live. Special attention should be given to communication via telemedicine, as virtual interactions with local health care professionals and parents may require a different approach and an additional skill set than traditional telephone or in-person interactions.

In addition to training, a structured evaluation method should be developed to measure the impact of TeleNeo (step 2.5). Both clinical and organizational outcomes can be considered, including patient outcomes, user experiences, staff workload, and financial aspects. Evaluations can be planned at prespecified time points, while allowing for adjustments as the program develops. The tools and examples included in Multimedia Appendix 4, “evaluation methods,” can serve as a guide.

Finally, contractual, procurement, legal, and financial aspects must be completed before go-live. Beyond establishing a contract for providing the telemedicine service and data sharing (if needed), programs should define a sustainable financial model for their service (step 2.6). Several contractual structures are possible. First, for agreements with the technical provider, organizations may choose between purchasing the equipment (which often requires higher upfront investments but lower long-term operational costs) or leasing hardware and software. Second, hardware and software access can be contracted as annual licensing for 24/7 availability or per-consultation fees. Third, financial agreements between hospitals may also vary: some collaborate without interhospital billing, while others use annual service fees to support readiness and staffing, or per-consultation payment models that align costs with actual use. Insurance payment for consultations varies by country. Early involvement of procurement, legal, and financial experts ensures regulatory compliance and supports the long-term financial sustainability of the TeleNeo program. Furthermore, involvement of clinical engineering/maintenance could be necessary during contractual phases to ensure that service and maintenance arrangements are appropriate (whether provided in-house or by the vendor).

### Section 3: Program Go-Live and Beyond

Section 3 consists of 3 steps that focus on launching, monitoring, and evaluating the TeleNeo program. This section uses the right side of Figure 3, “Prepare your implementation strategy and go live!”.

Prior to the program go-live, everyone involved must be notified of the date and time the new TeleNeo program will become available. It is important to use the preferred communication strategies and channels to inform, engage, and prepare the relevant stakeholders (step 3.1). Preexisting communication channels can be used to inform all stakeholders, as appropriate.

An appropriate evaluation and monitoring method is needed for quality assurance and to facilitate continuous improvement of the TeleNeo program. Monitoring should focus on use, program performance (eg, process metrics), patient impact, required assessment benchmarks, users’ needs and wishes (step 3.2). Once the program is established, it can be helpful to work with information technology staff to automate TeleNeo program metrics. It is often possible to incorporate automatic usage registrations into TeleNeo technologies so that service activations are automatically sent to program leadership on a weekly or daily basis to provide real-time data on TeleNeo usage. Furthermore, as the organization looks to improve, sustain, and potentially scale the TeleNeo program, a manager or administrator should be identified to lead program updates and improvements.

When telemedicine consultation frequencies are low, the program team should explore ways to ensure that current and newly hired staff remain familiar with TeleNeo. This can be achieved by having recorded training sessions available for staff to watch (eg, during onboarding) and/or implementing simulation training tailored to participants’ needs (step 3.3). When using simulation-based training, it is essential to identify the necessary resources, technologies, and scenarios [6].

## Discussion

### Purpose and Benefits of the TeleNeo Toolkit

The TeleNeo toolkit provides health care institutions with a practical guide to implementing telemedicine in neonatal care. Based on multinational experience, the toolkit helps teams identify care gaps amenable to a telemedicine solution and navigate the implementation process from start to finish. By combining practical tools and templates, the toolkit fosters multidisciplinary collaboration to enable the effective and sustainable integration of TeleNeo into clinical practice.

### Comparison With the Literature

Existing clinical telemedicine studies highlight persistent challenges in achieving consistent clinical impact across tele-intensive care unit programs and tele-emergency care models [25] and telemedicine initiatives in pediatrics and maternal-fetal medicine [26]. These studies emphasize the importance of technical reliability, rapid decision-making support, and remote specialist involvement. However, they do not sufficiently address the developmental, relational, and family-centered dimensions that are central to technological innovations in neonatal care, nor the highly interprofessional workflows characteristic of neonatal units. This tutorial adds to the clinical literature by synthesizing these important aspects of the innovation (eg, Multimedia Appendix 5), while placing them into real-life decision-making, including budgeting and relational arguments.

In parallel, implementation frameworks such as NASSS [10] and CFIR [17] underline the risk of nonadoption when innovations are not aligned with local context, stakeholder needs, and organizational readiness, and aim to guide users in identifying barriers specific to their implementation. The barriers for implementation of TeleNeo, presented and integrated into the steps of this toolkit, are based on these implementation frameworks combined with the experiences of the research team. By presenting case-specific barriers and advice to overcome these, this toolkit aims to enhance the implementation process by saving the implementing institution time needed to explore these themselves. More specific, to address the challenge of nonadoption of technological innovations in health care [10], the TeleNeo toolkit actively encourages stakeholders to co-design their own implementation pathway. By offering a structured, step-by-step, and visually guided process, the toolkit promotes a sense of ownership among users [27,28]; this is a factor that has been demonstrated to be essential for successful implementation. Stakeholders can incorporate their own experiences into the design process to ensure it aligns with local workflows, policies, and levels of technical literacy.

Current telehealth toolkits and guidelines, such as the “AMA (American Medical Association) Telehealth Implementation Playbook” [29] and the “WHO Telemedicine Guidelines” [9], provide broad, adaptable recommendations planned for diverse health care settings. Their wide scope is a strength for scalability but limits their applicability to specialty-specific workflows. They mainly focus on technical and organizational aspects and do not provide detailed guidance tailored to neonatal care pathways or family-centered care. Similarly, implementation process models, such as the knowledge-to-action model [30], offer valuable scientific structure for planning and evaluating implementation success. Yet they often remain conceptual and require translation into practical, context-specific steps before they can be applied effectively in clinical settings.

The TeleNeo toolkit addresses these gaps by offering specialty-specific, step-by-step guidance for the implementation of TeleNeo. It translates evidence-based frameworks into actionable processes and integrates insights from multinational real-world experience, enhancing its relevance across different health care systems. The ready-to-use templates and checklists support teams in adapting the implementation process to their local context, while maintaining fidelity to core principles of neonatal care and telehealth integration.

The visual representation of the toolkit facilitates communication across multidisciplinary teams and functions as a boundary object [31], enabling different stakeholder groups to develop a shared understanding despite diverse professional backgrounds. Boundary objects have been described as effective, strategic tools for aligning perspectives and establishing a common language in complex processes [32]. This approach supports users in reflecting on their current practices and systematically identifying opportunities for improvement, thereby fostering adoption and long-term sustainability.

By combining structured implementation logic with neonatal-specific considerations such as family-centered care, multidisciplinary collaboration, and developmental needs, the toolkit complements both general telehealth guidelines and theoretical implementation frameworks.

### Strengths and Limitations

The TeleNeo toolkit offers several strengths that make it a practical and reliable resource for TeleNeo implementation. It is grounded in real-world experience from different international settings and reflects challenges and successes encountered in clinical practice. Its step-by-step structure, templates, checklists, and visual elements translate complex tasks into concrete, actionable steps and support communication across professional groups. These features collectively strengthen the toolkit’s usability and contribute to a more consistent, coordinated, and sustainable integration of TeleNeo into neonatal care.

Some limitations of the toolkit remain. The toolkit is based primarily on experiences from high-resource health care systems, which may limit its adaptability in low-resource settings without modification. Furthermore, while the toolkit provides structured guidance, successful implementation still depends on local leadership, organizational readiness, and available technical infrastructure, all of which vary across institutions.

### Future Perspectives

In conclusion, the TeleNeo toolkit offers a practical, context-sensitive, and experience-based resource that supports health care institutions in implementing TeleNeo effectively and sustainably, while acknowledging that successful adoption ultimately depends on local conditions, leadership, and system readiness. We present this tutorial as the learning from our positive collective experiences and invite readers for future debate and mutual learning. We also invite readers to evaluate the implementation toolkit across diverse real-life health care settings, including its usability, adaptability, and impact on adoptions and sustainability of telehealth innovations.

## Supplementary material

10.2196/90592 Multimedia Appendix 1 Prior versions of the toolkit.

10.2196/90592 Multimedia Appendix 2 Workshop templates and feedback.

10.2196/90592 Multimedia Appendix 3 Stakeholder checklist.

10.2196/90592 Multimedia Appendix 4 Evaluation methods.

10.2196/90592 Multimedia Appendix 5 TeleNeo technology selection.

## Abbreviations

AMA: American Medical Association
CFIR: Consolidated Framework for Implementation Research
HCPs: health care providers
MAST: Model for Assessment of Telemedicine
MC: Medical Center
NASSS: nonadoption, abandonment, scale-up, spread and sustainability
NICU: neonatal intensive care unit
RE-AIM: Reach, Effectiveness, Adoption, Implementation, and Maintenance
TeleNeo: TeleNeonatology
WHO: World Health Organization
