# Supplementary material for: Implementing Telemedicine for Neonatal Care: Tutorial on a Practical Toolkit Based on Multinational Experience
Source: J Med Internet Res. 2026 Sep 30;28:e90592. doi: 10.2196/90592 (PMC13626644; doi:10.2196/90592)
Supplement: Supplementary file 1 [file jmir-v28-e90592-s001.pdf]

Version 1

Prior versions of the toolkit

TeleNeo Implementation Toolkit		Tools and activities
**Section 1 – Develop your TeleNeo Program**		
**Identify the gap**	Which specific clinical, educational, or organizational challenges and barriers (e.g., technology, policies, internal vs. external activities) currently exist in your NICU that could be addressed through telemedicine?<br><br>What are the key activities and interactions in your current neonatal care pathway, who is involved in each, and what outcomes are associated with these interactions?	• Ask parents, nurses, physicians for their thoughts on challenges and room for improvement<br>• Fill in the care pathway template in attachment A to help find potential for TeleNeo
**Identify stakeholders**	Who are the key stakeholders involved in inpatient and outpatient care, family communication during NICU admission, and organizational change processes (legal, financial, technical), and how influential are they?<br><br>What are the needs, wishes, and preferred ways of engagement of these stakeholders, and how can we effectively involve them considering their available time and accessibility?	• Start with the stakeholder list in attachment B and fill in step 1 of the *TeleNeo program template* in attachment C.<br>• Use stakeholder frameworks, such as salience framework, to help with determining the level of influence and dependence of specific stakeholders
**Plan TeleNeo program**	Which individuals or groups will be directly affected by the implementation of telemedicine, what is our desired goal (future vision), and what existing evidence supports this goal?<br><br>What specific outcomes and outputs are needed to optimize the process, which stakeholder needs must be prioritized, and how can we align our vision with the stakeholders' expectations?	• Interview key-stakeholders<br>• Search literature for evidence on TeleNeo impact<br>• Discuss first ideas with key-stakeholders to create a shared idea of the innovation<br>• Fill in step 2, 3, 4 and 5 of the *TeleNeo program template* in attachment C.
**Determine evaluation methods**	Which measures (clinical impact, experience, costs) and feedback points do we want to evaluate, how often and for how long should evaluation and follow-up be conducted, and which stakeholders need to be involved?	• Search literature for methods used to evaluate TeleNeo program.<br>• Suggested outcomes frameworks: MAST, RE-AIM, and by Proctor et al.<br>• For examples and explanations of evaluation methods for purposes see attachment D

	How can we organize the evaluation (individual vs. group assessments), who is responsible for follow-up and process adaptation, how can we assess the value of implementation, and what resources are needed?	
**Section 2 – Prepare TeleNeo Program Implementation**		
**Determine technological requirements**	Which existing infrastructures, platforms, or protocols can support telemedicine, what technological systems are already available in our hospital, and what additional developments are needed?<br><br>Which telemedical system fits our clinical setting (e.g., WIFI, space requirements), what technological changes are needed at each step, and who can support us internally in identifying the right technological solutions?	• Ask advice from technological experts in the hospital.<br>• Fill in step 6 of the *TeleNeo program template* in attachment C
**Explore costs and finances**	What investments are required and what funding options are available (e.g. research or innovation budgets)<br><br>What is the impact of TeleNeo program on structural costs? What are ongoing expenses, how does the program influences production incomes and what are available reimbursement structures?	• Ask advice from financial controllers<br>• Create a budget and preliminary business case<br>• Fill in step 8 of the *TeleNeo program template* in attachment C
**Explore barriers for implementation**	What physical and mental barriers might we face when implementing telemedicine, and what support or facilitators could help us overcome them?<br><br>How can we ensure clinical safety, data protection, and compliance with ethical standards during the implementation, and what steps are needed to achieve our future vision?	• Frequently used frameworks to explore barriers are: NASSS and CFIR. Both include a toolkit to guide you through the framework<br>• Ask advice from privacy, legal and ethical experts<br>• Fill in the barriers in the *implementation timeline template* in attachment E
**Create workflow overview aligned with stakeholder**	What changes need to be made to the current workflow, and which care providers will take on new or different tasks?<br><br>How can we balance new telemedicine tasks with existing duties, and who will be responsible for	• Discuss with relevant healthcare professionals from both hospitals<br>• Do try-out sessions<br>• Time current workflows and new workflows to compare workload

	monitoring and ensuring that the implementation goes smoothly?	• If necessary, adjust step 5 of the *TeleNeo program template*<br>• Make clear workflow overview
**Define the implementation timeline**	In how many weeks, months, or years do we want to achieve our final goal, and which steps and milestones need to be completed within which timeframes?<br><br>Who needs to be involved at each stage of the timeline, what resources are required when, and how can we allocate these resources effectively?	• Fill in the three upper rows of the *implementation timeline template* in attachment E
**Design training program**	How many training sessions are needed for different user groups, and in what format (digital, physical, VR; group vs. individual) should the training sessions be delivered?<br><br>Who should deliver the training (professional trainer vs. peer colleague), and how can we provide accessible training materials for follow-up questions or ongoing education?	• When possible, integrate with existing training sessions<br>• Collaborate with educational experts<br>• Supplement the *to do's* in the *implementation timeline template* in attachment E
**Section 3 – Go Live**		
**Plan both methods and moments for the evaluation session**	How and where should we gather and evaluate feedback (e.g. digital, anonymous, in-person), who should be responsible for the process, and which stakeholder groups should we prioritize for meaningful input?<br><br>How often should evaluation sessions take place, which session types (e.g. simulation vs. clinical) should be included, and should the frequency or format of evaluations change over time?	• Have a look at the evaluation methods in attachment D - which methods would be the most suitable?<br>• Fill in step 7 in the *TeleNeo program template* in attachment C
**Go live with the program**	What communication strategies and channels can we use to ensure that all relevant staff and stakeholders are informed, engaged, and prepared for the program launch?<br><br>How can we effectively communicate and promote the program launch to ensure that all relevant staff, stakeholders, and users are aware and prepared?	• Use existing communication channels, like newsletters, screensavers or weekly meetings

Version 1

**Ongoing monitoring of program performance**	How can we track and visualize the program's performance over time, and what indicators or benchmarks should we use to assess whether it works well?<br><br>How can we systematically and ideally automatically register the use of TeleNeo (e.g., consultations) to support continuous performance monitoring?	• Ask technicians for advice on how to automatically track and visualize the TeleNeo program<br>• Often the technical companies have automatic registration services available
**Ongoing simulation training**	When and how should we offer additional simulation training, and who should participate in these sessions?<br><br>What resources, technologies, and scenario types are needed to ensure meaningful and targeted simulation training opportunities?	• Define a set of key training scenarios and create a standardized simulation script for each (including objectives, who is involved, and what equipment or setting is needed)
**Update program and workflow based on feedback**	How can we gather and interpret the needs and wishes of users and stakeholders in a way that they can be translated into concrete action points?<br><br>Who needs to be involved in updating the program and workflow such as designers, stakeholders, user groups, or initiative takers and how can we organize their collaboration?	• Collaborate and check with key stakeholders when updating the program

1. Who is involved and will be affected by implementing the TeleNeo?

E.g. patients, nurses, family, other health providers

Value
Patient

Value
Healthcare professional

Value
Hospital

Value
...

Value
...

3. What is the added value of implementing TeleNeo?

E.g. for the patient, parent, healthcare professional, hospital and...

2. TeleNeo purpose

E.g. with TeleNeo, we want to improve care for neonates by ...

8. What are the investments and structural costs connected to implementing the TeleNeo?

E.g. what funding source can you reach out to and who do you need to involve?

Investments				*Structural Costs*			
Source	Who	Source	Who	Source	Who	Source	Who
Source	Who	Source	Who	Source	Who	Source	Who

4. What needs need to be met during implementation?

E.g. support, safety, ease

5. Which TeleNeo activities need to be done to reach the TeleNeo purpose?

E.g. consultations, resuscitation, handover etc.

Activity 1

Activity 2

Activity 3

Activity 4

6. What are the technical, safety and security requirements for implementation per activity?

7. How can the impact per activity be evaluated?

	Before implementation	Pilot implementation	Sustained implementation	FUTURE
**Timespan**				
**Goal per step**	Create a detailed implementation plan containing easy to follow to dos	Start implementation and test initial assumptions	Optimal embedding into care pathways and work routines, with support from relevant stakeholders	
**Stakeholder in focus**<br>*Stakeholders connected to each other and the activities in every step.*<br>*E.g. providers, patients, clients etc.*				
**Barriers to overcome**<br>*E.g. technological, organisational, human, economic, political, etc.*				
**To do's**<br>*E.g. training activities, consultations, documentation, evaluation, collaborations, implementation strategies, presentations, informal talks, demo sessions*				**TeleNeo Purpose**<br>..............................<br>..............................<br>..............................<br>..............................

Version 2

TeleNeo Implementation Toolkit		
**Section 1 – Design your TeleNeo Program** (use template in attachment A to guide you through the steps)		
**Action**	**Considerations**	**Examples of what you can do to answer the questions**
1.1<br><br>*Identify the gap*	• What specific clinical, educational, or organisational challenges exist in our NICU that could be addressed through telemedicine?<br>• Could telemedicine help to overcome the limitations of our care network's capabilities and/or resources?	Ask key-users (parents, nurses, physicians) for their thoughts on challenges and room for improvement.<br><br>Search literature for evidence on TeleNeo impact.
1.2<br><br>*Identify stakeholders – beneficiaries*	• Which stakeholders are involved in overcoming challenges during different stages of neonatal care?<br>• What are the needs and wishes of potential beneficiaries of the telemedicine intervention?<br>• How can we effectively involve and engage them, bearing in mind their availability, preferences and accessibility?	Interview key-users for insights in how to optimise the potential impact of TeleNeo from their perspective.<br><br>Discuss first ideas with key-stakeholders to create a shared idea of the innovation.
1.3<br><br>*Identify stakeholders – partners and influentials*	• Who are the key stakeholders involved in the healthcare delivery of telemedicine?<br>• Who needs to be involved in facilitating organisational and care pathway changes, and what influence do they have?<br>• How can we effectively involve and engage them, bearing in mind their availability, preferences and accessibility?	Use stakeholder frameworks (e.g. salience framework) to help with determining the level of influence and dependence of specific stakeholders.<br><br>Look at list of potential stakeholders in Attachment C.<br><br>Take the legal, financial and technical sides of organisational change into account.
**Iteration:** after talking with your stakeholders go back to your gaps and use those answers to define the next steps below		

1.4<br><br>*Determine TeleNeo activities*	• What contact occurs between healthcare providers during neonatal activities such as admissions?<br>• What current care activities can and should be performed using telemedicine?	Consider which care activities could be supported by TeleNeo technology.
1.5<br><br>*Define the telemedicine workflow*	• How do the current workflows change when using telemedicine?<br>• Who will be responsible for staffing the telemedicine service, monitoring its performance and ensuring it meets the desired standard?	Involve key-users (e.g. nurses, physicians).<br><br>Conduct site visits to gain an understanding of the impact of changes to the current workflow.<br><br>Consider your organisation's unique documentation requirements.
1.6<br><br>*Specify TeleNeo requirements*	• What functionality, technical performance, privacy, system integration and data storage requirements does the telemedicine hardware and software need to meet?<br>• What existing infrastructures, platforms, or protocols can support telemedicine?	Ask advice from specific experts (e.g. technology) in your health system.<br><br>Look for examples of telemedicine within your own hospital.
1.7<br><br>*Explore costs and finances*	• What funding options are available for initial costs (e.g. research or innovation budgets)?<br>• What are the structural TeleNeo program costs (licenses, technical requirements, staffing)?<br>• What is the impact of the TeleNeo program on patient-related incomes of the healthcare system and what reimbursement structures are available?	Ask advice from financial controllers.<br><br>Create a budget and preliminary business case

Version 2

**Section 2 – Prepare your implementation strategy** (use template in attachment B to guide you through the steps)		
**Action**	**Considerations**	**Examples of what you can do to answer the questions**
2.1 Engage users	• What do we need to create a shared future goal with key-users?	Give presentations and use informal talks with key-users.
2.2 Barriers for implementation	• What physical and mental barriers might we encounter when implementing telemedicine, and how can we overcome them with the right support and resources?<br>• What steps are needed to ensure clinical safety, data protection and compliance with ethical standards during implementation?	Frequently used frameworks to explore barriers are: NASSS and CFIR. Both include a toolkit to guide you through the framework.<br><br>Ask advice from privacy, legal and ethical experts.
2.3<br><br>*Define timeline*	• When do interim gatekeeping milestones need to be completed to achieve the target go-live date?<br>• Who needs to be involved at each stage of the timeline, and what resources are required when?	Use the timeline on attachment B to define the steps.
2.4<br><br>*Staff training program*	• How should training sessions be designed in terms of frequency, content and delivery method (professional or peer colleagues) and medium (digital, physical or VR; group or individual)?<br>• What training materials do we need for follow-up questions and/or ongoing education?<br>• How can the training programme be connected to changes in communication styles when using TeleNeo (e.g. digital vs phone, presence of parents)?	When possible, integrate with existing training sessions.<br><br>Collaborate with educational experts.<br><br>Include digital communication style into training.
2.5 Identify program evaluation metrics	• What process, outcome, cost, and experience measures should we evaluate?<br>• How do we gather and evaluate feedback from users (e.g. digital, anonymous, in-person, individually or in groups)?<br>• How can we monitor and improve the quality and performance of TeleNeo to ensure that the results are as desired and designed?	Search literature for methods used to evaluate TeleNeo program.<br><br>For examples and explanations of evaluation methods for purposes see attachment D.
2.6	• What legal contracts are necessary for the telemedicine program (e.g. data transfer agreements, collaboration agreements, legal responsibility of the patient)?	Involve legal and financial experts.

Legal and finances	• Who needs to approve the business case and financial impact of TeleNeo?	
**Section 3 – Go Live and Beyond** (continue with template in attachment B)		
**Action**	**Considerations**	**Examples of what you can do to answer the questions**
3.1<br><br>*Go live!*	• Which communication strategies and channels could we use to ensure that all relevant staff and stakeholders are informed, engaged, and prepared for program launch?	Use existing communication channels, like newsletters, screensavers or weekly meetings.
3.2<br><br>*Program monitoring and continuous improvement*	• How can we track and visualise the programme's performance over time, considering the needs and wishes of users and stakeholders?<br>• How can we systematically and ideally automatically register the use of TeleNeo to support continuous performance monitoring?<br>• Who needs to be involved in updating the programme and workflow, and how can we organise their collaboration (e.g. designers, stakeholders, users, initiators)?	Ask technicians for advice on how to automatically track and visualise the TeleNeo program.<br><br>Often the technical companies have automatic registration services available.<br><br>Collaborate and check with key stakeholders when updating the program.
3.3<br><br>*Ongoing training*	• When and how should we offer additional simulation training, and who should participate in these sessions?<br>• How can we introduce and prepare new staff for the telemedicine program?<br>• What resources, technologies, and scenario types are needed to ensure meaningful and targeted simulation training opportunities?	Define a set of key training scenarios and create a standardised simulation script for each (including objectives, who is involved, and what equipment or setting is needed).

1.1 What problems are we solving with TeleNeo?

E.g. with TeleNeo, we want to improve care for neonates by ...

1.2 Who will benefit from TeleNeo and how?

E.g. patient, parent, healthcare professional, hospital...

1.3 Who do you need when implementing TeleNeo?

E.g. nurses, technicians, financial advisors, surgeons

Iterate your findings
Is everyone included?

1.4 Which neonatal care activities do you want to perform using TeleNeo?

E.g. consultations, resuscitation, handover etc.

Activity 1

Activity 2

Activity 3

Activity 4

1.5 What will change in current workflows?

1.6 What are the technical, safety and security requirements for implementation per activity?

1.7 What are the investments and structural costs connected to implementing the TeleNeo?

E.g. what funding source can you reach out to and who do you need to involve?

Investments

Source	Who
Research Funding	*Grant proposal writing*

Structural Costs

Source	Who
Reimbursement for Tele-Consultations	*Insurance company/ hospital insurer*

Prepare Implementation

2.1 How can we align our plan with the stakeholders' expectations?

2.2 What are barriers for implementation of your program?
E.g. technological, organisational, human, safety economic, political, etc.

2.3 How do you integrate TeleNeo within your current workflows?

Changes and task differentiation

Balance with existing duties and responsibility

2.4 When do you want the TeleNeo program to be up and running?

2.5 How can you prepare and train all key-users for TeleNeo?

Training sessions
Who participates and provides training, when and what format?

Training materials
What, how accessible and how continued?

Communication style
Is it changing during the use of the TeleNeo?

2.6 How can you effectively measure the impact of the TeleNeo program?

Measures and feedback points

Responsibility

Gathering and Evaluating Feedback

Timeframe

2.7 What contracts and official approvals are needed before implementation?

Go Live!

3.1 Let everyone know the TeleNeo project has started
E.g. communications strategies and channels, program launch, stakeholders involved

3.2 How can you monitor and evaluate the TeleNeo program?

Track and visualize performance

Register the use

Audience for results

Add evaluation to the timeline!

3.3 How do you ensure exposure to TeleNeo when TeleNeo consultation frequencies are low?

3.4 What improvements are necessary to ensure smooth integration into workflows and reaching the aimed impact?

Gather Needs and Wishes

People involved in updating program and workflow
