# Supplementary material for: Implementing Telemedicine for Neonatal Care: Tutorial on a Practical Toolkit Based on Multinational Experience
Source: J Med Internet Res. 2026 Sep 30;28:e90592. doi: 10.2196/90592 (PMC13626644; doi:10.2196/90592)
Supplement: Supplementary file 2 [file jmir-v28-e90592-s002.pdf]

Template 1 Develop your TeleNeo Program							
	What problems are we solving with TeleNeo?	*Who will benefit from TeleNeo and how?*	*Who do you need when implementing TeleNeo?*	*Which neonatal care activities do you want to perform using TeleNeo?*	*What will change in current workflows?*	*What are the technical, safety and security requirements for implementing per activity?*	*What are the investments and structural costs connected to implementing the TeleNeo?*
**P1**	Initail neonatal care, consultations, assessment of infections	Patients, parents, doctors, nurses, midwifes, resources	Doctors, nurses, patients, IT, insurance group, head of the hospital	Interdisciplinary consultations	On-site support ensures that the team can reach the delivery room more rapidly, reducing transfers, children remain with their parents, leading to greater patient satisfaction and an increased number of beds available in the NICU, faster diagnoses, earlier initiation of therapy	The setup includes permanently installed cameras in the initial care room, telemedicine support in the resuscitation room, fixed tablets on the ward, and tablets at patients' homes with dedicated apps. Data protection, liability issues, and whether sessions are recorded are carefully considered, alongside efforts to standardize programs nationwide.	Writing proposal for grants, technical equipment, communication with insurenace groups
**P2**	Structured processes, efficient use of resources, and access to expertise in remote hospitals, consultations for parents, teamwork throughout different disciplines	Patients, parents, team	All professions, stakeholders, politicians	Direct consultations with surgeons, including during procedures in the operating room	Additional workload, time savings, avoidance of unnecessary transfers, improved coordination	Data protection, liability, IT, employee safety, works council, consent form	Donations, research funding (grant proposal writing), reimbursement for teleconsultations (cost reduction through insurance groups)
**P3**	Surgical consultations, interdisciplinary work	Patients, parents, team, hospital	Nurses, IT team, legal experts, telemdical providers	Planing of transfers, consultations during surgery or before surgery	More communication	No answer	Research funding (writing proposals), reimbursement for teleconsultations (cost reduction through insurance groups)
**P4**	Reducing transfers	Patient, family/parents, team at the hospital, community-based physicians, hospital	Doctors, nurses, IT team, legal experts, patients and parents, telemedical provider	Neonatal initail care, PICU, post-discharge consultations	Key aspects include the workplace setup, training programs to develop IT skills, a clear timeline, structured communication, final decision-making authority, follow-up care, and general documentation practices	Room for telemedicine	Research funding (writing proposals), reimbursement for teleconsultations (cost reduction through insurance groups)
**P5**	Initail neonatal care in remote hospitals without a neonatology ward	Patients, parents, health care providers	No answer	Consultations with radiology and for initial care, consultation during BLS, NLS, ALS, virtual consultations in general, transfers	Different schedules, IT, stable internet connection, different team structure,	Enough personnel, stable internt connection	No answer
**P6**	Improve teamwork, rapis intensive care consultations, clinical outcomes, improve decision making	Patients, parents, doctors	First the doctors as they might feel "exposed" and "confronted" with the team dynamics and decision making versus solo/individual decision making	No answer	No answer	Innovation, capture intervention/agree on responsibilities, future orientation/what is the future impact/expectation	No answer
**P7**	Initial neonatal care	Patients	Neonatology department, docotrs, nurses, midwifes, Anaesthesiology, legal experts, pediatrcians	Neonatology, transfers, interdisciplinary work	New technical equipment	No answer	No answer

Patienten/Eltern

Ärzte

Pflege

Hebammen

Ressourcenschonung

2. Wer soll von Telemedizin profitieren?

z.B. Patient:innen, Eltern, Gesundheitseinrichtungen,...

1. Welches Problem soll mit Telemedizin gelöst werden?

(1) Unterstützung Erstversorgung Geburtsklinik
(2) Beurteilung leichte Infektionen/ANS Wochenstation
→ (1) Bessere Erstversorgung bis Neo vor Ort
(2) Kinder können ggf. bei Mutter bleiben
beide: spart Transporte/Fahrten

z.B. Mit Telemedizin wollen wir die Versorgung verbessern von...

3. Wer muss bei der Implementierung von Telemedizin miteinbezogen werden?

z.B. Patient:innen, Pflegekräfte, Familie,...

- Ärzte
- Pflege
- Patienten/Eltern
- IT
- Betriebsrat
- Krankenhausleitung
- Krankenkassen

Iterieren Sie ihre Ergebnisse

4. Welche Versorgungsbereiche sollen von Telemedizin unterstützt werden?

z.B. Konsultationen, Reanimationen, Transfer,...

Aktivität 1	Aktivität 2	Aktivität 3	Aktivität 4
s.o. (1)	s.o. (2)	(3) sozialmed. Nachsorge?	Konsultation Dermatologe (4)

5. Was wird sich im jetzigen Workflow ändern?

Aktivität 1	Aktivität 2	Aktivität 3	Aktivität 4
Unterstützung Kreißsaal-Team bis Neo vor Ort	weniger Transporte, Kinder bei Eltern → Patientenzufriedenheit, ~~[illegible]~~ mehr Betten auf NICU für kranke NG/FG	spart evtl. Hausbesuche? → Personal-Ressourcen, Klinik-Schnittstelle	ggf. schnellere Diagnose/Therapie?

6. Welche technischen (1), Sicherheits- (2) und Datenschutzanforderungen (3) sind für die Umsetzung der einzelnen Aktivitäten notwendig?

Aktivität 1	Aktivität 2	Aktivität 3	Aktivität 4
(1) fest installierte Kamera in Erstversorgungsraum + Leitung	entweder über Rea-Raum-Device oder Tablet-based auf Station	Tablet-based/Apps? zu Hause bei Pat.	Tablet-based? Rechner-based.

(2+3) Datenschutz; Haftungsfragen; → Aufzeichnung ja/nein/; Evaluation; Vereinheitlichung der Programme landesweit!

ad 1) Personalschulung an der neuen Technik!

7. Welche Investitionen und Kosten sind mit der Umsetzung des TeleNeo Programms verbunden?

z.B. Welche Finanzierungsmöglichkeiten gibt es (z.B. Forschungs- oder Innovationsbudgets)?

Investitionen

Quelle	Wer
Stiftungen z.B. [illegible]-Stiftung	Anträge schreiben
Forschungsförderung	Schreiben von Förderanträgen
Kostenerstattung für Telekonsultationen	Versicherungsgesellschaft

Kosten

Quelle	Wer
technische Ausstattung	
Personal aufstocken?	
auf lange Sicht hoffentlich Einsparungen!	

1. What problems are we solving with TeleNeo?

Resourcenschonung / unnötige Transporte / Trennung Eltern
Expertise in Randzeiten
Strukturierung Prozesse / medizinische Prozesse

E.g. with TeleNeo, we want to improve care for neonates by ...

2. Who will benefit from TeleNeo and how?

E.g. patient, parent, healthcare professional, hospital...

- Kinder
- Eltern
- Team
- Kommunikation
- Vernetzung Standorte
- Patientenacquise Referral-Zentrum
- Vernetzung Team
- Education Zentren + Team
- Nachsorge
- Weiterbildung
- Ethik

3. Who do you need when implementing TeleNeo?

E.g. nurses, technicians, financial advisors, surgeons

Alle Berufsgruppen Hebammen Geburtshelfer
Geldgeber
Politik

4. Which neonatal care activities do you want to perform using TeleNeo?

E.g. consultations, resuscitation, handover etc.

Activity 1	Activity 2	Activity 3	Activity 4
Reanimation	Konsil Subspezialitäten	Transfer	Bereitschaftsdienst

5. What will change in current workflows?

Mehrarbeit in Telemedizin-Keller	Zeitgewinn durch Vermeidung unnötiger Verlegung	Koordination Abläufe in Zentrum	

6. What are the technical, safety and security requirements for implementation per activity?

Datenschutz	IT-Sicherheit	Betriebsrat	Consent?
Haftbarkeit	Mitarbeitersicherheit		

7. What are the investments and structural costs connected to implementing the TeleNeo?

E.g. what funding source can you reach out to and who do you need to involve?

Investments

Source	Who	Source	Who	Source	Who
Spender		Science Funding		Kassen Leistung	
Research Funding	Grant proposal writing			Reimbursement for Tele-Consultations	Insurance company/ hospital insurer

Structural Costs

Source	Who	Source	Who	Source	Who
		Science		Kasse	

1. Welches Problem soll mit Telemedizin gelöst werden?

Chir. Konsultation bei Neugeborenen

Kinderkard. Mitbeurteilung

z.B. Mit Telemedizin wollen wir die Versorgung verbessern von...

2. Wer soll von Telemedizin profitieren?

z.B. Patient:innen, Eltern, Gesundheitseinrichtungen,...

- Patientinnen
- Eltern: • wohnortnahe Betreuung
  • Sicherheit / Vertrauen ↑
- Versorgungsteam vorOrt
  • Mehr Sicherheit
  • Bessere Qualität
- Versorg. Team bei Fernbehandlung
  - Pat. Allokation
  - frühzeitige Verlegung / Zurückverl.
- ~~[illegible]~~:
  Krankenkassen:
-

3. Wer muss bei der Implementierung von Telemedizin miteinbezogen werden?

z.B. Patient:innen, Pflegekräfte, Familie,...

Netzwerk Strukturen in Deutschland

Datenschutz / Rechtl. Regelungen

Krankenkassen

Kaufmännische Leitungen Träger Fern → Latf. KH

Pflege

4. Welche Versorgungsbereiche sollen von Telemedizin unterstützt werden?

z.B. Konsultationen, Reanimationen, Transfer,..

Aktivität 1	Aktivität 2	Aktivität 3	Aktivität 4
Konsultation / OP Indik.?	Echokardiol.		
→ Transportplanung	Mitbeurteilung		

5. Was wird sich im jetzigen Workflow ändern?

Mehr Kommunikation

6. Welche technischen, Sicherheits- und Datenschutzanforderungen sind für die Umsetzung der einzelnen Aktivitäten notwendig?

7. Welche Investitionen und Kosten sind mit der Umsetzung des TeleNeo Programms verbunden?

z.B. Welche Finanzierungsmöglichkeiten gibt es (z.B. Forschungs- oder Innovationsbudgets)?

Investitionen

Quelle	Wer	Quelle	Wer	Quelle	Wer

Kosten

Quelle	Wer	Quelle	Wer
Forschungsförderung	*Schreiben von Förderanträgen*	*Kostenerstattung für Telekonsultationen*	*Versicherungsgesellschaft*

Quelle	Wer	Quelle	Wer	Quelle	Wer

Patienten

Familie (Provider to Patient)

Niedergelassene

Teams vor Ort / Rückverlegung

Kostenträger

2. Wer soll von Telemedizin profitieren?

z.B. Patient:innen, Eltern, Gesundheitseinrichtungen,...

3. Wer muss bei der Implementierung von Telemedizin miteinbezogen werden?

z.B. Patient:innen, Pflegekräfte, Familie,...

Pflege, IT, Kaufmänner, Hersteller, Ärzte, Anwälte

Patient

1. Welches Problem soll mit Telemedizin gelöst werden?

- Verlegungen vermeiden / Vor Ort lösen
- Hilfe bei Zentralisierung

z.B. Mit Telemedizin wollen wir die Versorgung verbessern von...

Iterieren Sie Ihre Ergebnisse

4. Welche Versorgungsbereiche sollen von Telemedizin unterstützt werden?

z.B. Konsultationen, Reanimationen, Transfer,..

Aktivität 1	Aktivität 2	Aktivität 3	Aktivität 4
Erstversorgungsraum	Pico / ITS	Schockraum	Konsile nach Extern / Niederlassung

5. Was wird sich im jetzigen Workflow ändern?

Arbeitsplatz	Zeitplan	wer hat das letzte Wort?	Dokumentation (beidseits)
Schulung / Skills IT	Kommunikation	Follow-up	↳ Patient = ? ohne?

6. Welche technischen, Sicherheits- und Datenschutzanforderungen sind für die Umsetzung der einzelnen Aktivitäten notwendig?

- Raum
- Endgeräte

7. Welche Investitionen und Kosten sind mit der Umsetzung des TeleNeo Programms verbunden?

z.B. Welche Finanzierungsmöglichkeiten gibt es (z. B. Forschungs- oder Innovationsbudgets)?

Investitionen

Quelle	Wer
Tele Nova	
Personal	
Stiftung	
Forschungsförderung	Schreiben von Förderanträgen

Kosten

Quelle	Wer
Kostenerstattung für Telekonsultationen	Versicherungsgesellschaft

1. Welches Problem soll mit Telemedizin gelöst werden?

z.B. Mit Telemedizin wollen wir die Versorgung verbessern von...

Wir möchten die Erstversorgung von Neugeborenen im Kreißsaal verbessern, wo kein Pädiater/Neonatologe primär vor Ort ist.

2. Wer soll von Telemedizin profitieren?

z.B. Patient:innen, Eltern, Gesundheitseinrichtungen,...

Patient:innen + Eltern
Team vor Ort sowie
das Neo-Team, was
Beratung/Unterstützung anbietet.

3. Wer muss bei der Implementierung von Telemedizin miteinbezogen werden?

z.B. Patient:innen, Pflegekräfte, Familie,...

Geschäftsführung / Ärztl. Direktor
Ärztl. + Pflegeteam, Techniker/IT-Team
Patient:innen + Eltern

4. Welche Versorgungsbereiche sollen von Telemedizin unterstützt werden?

z.B. Konsultationen, Reanimationen, Transfer,...

Aktivität 1	Aktivität 2	Aktivität 3	Aktivität 4
Konsultationen (Sonographie, Radiologie, Erstversorgung)	Reanimationsunterstützung	Virtuelle Visiten	Verlegung auf MCU

5. Was wird sich im jetzigen Workflow ändern?

Aktivität 1	Aktivität 2	Aktivität 3	Aktivität 4
Zeit planen, Technik/IT entsprechend aufbauen	Technik/IT/stabiles WLAN<br>Team mit Know how anwesend	Technik/IT<br>Erfahrenes Team anwesend	=<br>=

6. Welche technischen, Sicherheits- und Datenschutzanforderungen sind für die Umsetzung der einzelnen Aktivitäten notwendig?

- Ausreichend Personal anwesend ⟷ (gilt für alle Aktivitäten)
- WLAN/IT-Supportteam

7. Welche Investitionen und Kosten sind mit der Umsetzung des TeleNeo Programms verbunden?

z.B. Welche Finanzierungsmöglichkeiten gibt es (z.B. Forschungs- oder Innovationsbudgets)?

Investitionen

Quelle	Wer	Quelle	Wer	Quelle	Wer

Kosten

Quelle	Wer	Quelle	Wer
Forschungsförderung	Schreiben von Förderanträgen	Kostenerstattung für Telekonsultationen	Versicherungsgesellschaft

Quelle	Wer	Quelle	Wer	Quelle	Wer

1. Welches Problem soll mit Telemedizin gelöst werden?

-> intervision instantly
improve teamwork / improve decision making / Clinical ~~out~~ outcome

z.B. Mit Telemedizin wollen wir die Versorgung verbessern von...

2. Wer soll von Telemedizin profitieren?

z.B. Patient:innen, Eltern, Gesundheitseinrichtungen,...

- Patienten -
- Eltern /
- Ärzte /

3. Wer muss bei der Implementierung von Telemedizin miteinbezogen werden?

z.B. Patient:innen, Pflegekräfte, Familie,...

- First the doctors as they might feel "exposed" and "confronted" with team dynamics and decisions versus solo / individual decision making /
-

4. Welche Versorgungsbereiche sollen von Telemedizin unterstützt werden?

z.B. Konsultationen, Reanimationen, Transfer,..

Aktivität 1	Aktivität 2	Aktivität 3	Aktivität 4

5. Was wird sich im jetzigen Workflow ändern?

~~[illegible]~~ /

6. Welche technischen, Sicherheits- und Datenschutzanforderungen sind für die Umsetzung der einzelnen Aktivitäten notwendig?

+ First address the vulnerability / innovation = changed behaviour ->
+ Capture interaction / agree on responsibilities
+ future orientation : what is the future impact / expectation shortage situation etc.
~~[illegible]~~

7. Welche Investitionen und Kosten sind mit der Umsetzung des TeleNeo Programms verbunden?

z.B. Welche Finanzierungsmöglichkeiten gibt es (z. B. Forschungs- oder Innovationsbudgets)?

Investitionen

Quelle	Wer	Quelle	Wer	Quelle	Wer

Quelle	Wer
Forschungsförderung	Schreiben von Förderanträgen

Kosten

Quelle	Wer
Kostenerstattung für Telekonsultationen	Versicherungsgesellschaft

Quelle	Wer	Quelle	Wer	Quelle	Wer

1. Welches Problem soll mit Telemedizin gelöst werden?

Erstversorgung in der Peripherie
Vorbereitung Abholung
" Rückverlegung

z.B. Mit Telemedizin wollen wir die Versorgung verbessern von...

2. Wer soll von Telemedizin profitieren?

z.B. Patient:innen, Eltern, Gesundheitseinrichtungen,...

Patientinnen

3. Wer muss bei der Implementierung von Telemedizin miteinbezogen werden?

z.B. Patient:innen, Pflegekräfte, Familie,...

CA Neo + Geb.
OÄ Neo
Pflege
Hebamme
Anästhesiste
Juristen
Kinderärzte

iterieren Sie Ihre Ergebnisse

4. Welche Versorgungsbereiche sollen von Telemedizin unterstützt werden?

z.B. Konsultationen, Reanimationen, Transfer,...

Aktivität 1	Aktivität 2	Aktivität 3	Aktivität 4
Neo-Rea Kreißsaal Neo	Neo Konsil Neo	Verlegung Neo, KCH, Augenklinik	Konsil peripher alle bet. Spezialitäten

5. Was wird sich im jetzigen Workflow ändern?

ersetzen Rufdienst nötig

6. Welche technischen, Sicherheits- und Datenschutzanforderungen sind für die Umsetzung der einzelnen Aktivitäten notwendig?

Aufnahmepapiere ändern
gesicherte Verbindungen
Kamera / Mikro vor Ort Tablet auf Station

7. Welche Investitionen und Kosten sind mit der Umsetzung des TeleNeo Programms verbunden?

z.B. Welche Finanzierungsmöglichkeiten gibt es (z. B. Forschungs- oder Innovationsbudgets)?

Investitionen

Quelle	Wer

Kosten

Quelle	Wer
Forschungsförderung	Schreiben von Förderanträgen
Kostenerstattung für Telekonsultationen	Versicherungsgesellschaft
