# Supplementary material for: Implementing Telemedicine for Neonatal Care: Tutorial on a Practical Toolkit Based on Multinational Experience
Source: J Med Internet Res. 2026 Sep 30;28:e90592. doi: 10.2196/90592 (PMC13626644; doi:10.2196/90592)
Supplement: Supplementary file 3 [file jmir-v28-e90592-s003.pdf]

Stakeholder Checklist

- ❑ Patient and/or representatives
- ❑ Caregivers
- ❑ Medical team
  - ☐ Nurse
  - ☐ Nurse practitioner
  - ☐ Pediatrician
  - ☐ Neonatologist
  - ☐ Coordinator
  - ☐ Medical transport coordinator
  - ☐ Other…
- ❑ Other care team members
  - ☐ Dietician
  - ☐ Physiotherapist
  - ☐ Social work
  - ☐ Psychologist
  - ☐ Other…
- ❑ Other medical/surgical specialists
  - ☐ Cardiology
  - ☐ Genetics
  - ☐ Gynaecologist
  - ☐ Neurology
  - ☐ Ophthalmology
  - ☐ Surgery
  - ☐ Other …
- ❑ Technology and logistics
  - ☐ Medical technologist
  - ☐ Information and communication technology (ICT)- Expert
  - ☐ Data security expert
  - ☐ Privacy expert
  - ☐ Representative from technology vender (if used)
  - ☐ Medical transport coordinator
  - ☐ Other..
- ❑ Legal experts with focus on:
  - ☐ Medical licensing and the legal landscape
  - ☐ Inter-hospital contracting
  - ☐ Insurance companies
  - ☐ Other..
- ❑ Governance
  - ☐ Business analysist
  - ☐ Financial controllers
  - ☐ Medical management/board of departments/hospital (legal responsible)
  - ☐ Other…
