# Supplementary material for: Implementing Telemedicine for Neonatal Care: Tutorial on a Practical Toolkit Based on Multinational Experience
Source: J Med Internet Res. 2026 Sep 30;28:e90592. doi: 10.2196/90592 (PMC13626644; doi:10.2196/90592)
Supplement: Supplementary file 4 [file jmir-v28-e90592-s005.pdf]

TeleNeonatology Technology Selection

Below are questions to help you select a product for your teleneonatology program. These questions can be used to determine if your hospital system has a product that is suitable for tele-resuscitation or if a new product needs to be identified. Examples of potential technologies to use for tele-resuscitation are:

- Lower tech, e.g., portable tablets or phones with privacy-compliant video conferencing software
- Higher tech, e.g., mobile telemedicine cart with multiple cameras or fixed/mounted cameras
- Augmented reality, e.g., headsets for hands-free guidance

Domain	***Meets Requirements?***	***Priority?***	***Notes***
**Reliability**			
Uptime ≥ 99%	□ Yes □ No	□ High □ Low	
Clear process for rapid identification of technical issues	□ Yes □ No	□ High □ Low	
Clear process for rapid resolution of technical issues	□ Yes □ No	□ High □ Low	
**Usability**			
Remote consultant interface is simple and intuitive	□ Yes □ No	□ High □ Low	
Device type is appropriate for environment (cart, ceiling, wall-mount)	□ Yes □ No	□ High □ Low	
Device fits within the resuscitation space	□ Yes □ No	□ High □ Low	
Setup for community hospital team is quick and easy under high-stress conditions	□ Yes □ No	□ High □ Low	
If mobile: battery backup available with sufficient battery life	□ Yes □ No	□ High □ Low	
**Video and Audio Quality**			
Video quality allows for accurate infant assessment and confidence when guiding care	□ Yes □ No	□ Hig h □ Low	
Multiple camera angles available (to view infant, team, monitor, etc.)	□ Yes □ No	□ High □ Low	
Remote neonatologist can control camera views	□ Yes □ No	□ High □ Low	
Audio is clear, uninterrupted, and loud enough for both team and remote neonatologist	□ Yes □ No	□ High □ Low	
**Security and Privacy**			
Meets enterprise security requirements	□ Yes □ No	□ High □ Low	
Meets patient privacy requirements	□ Yes □ No	□ High □ Low	
Secure video recording available if needed	□ Yes □ No	□ High □ Low	
**Interoperability and Costs**			
Works on current IT infrastructure, or minimal upgrades required	□ Yes □ No	□ Hig h □ Low	
Integrates with electronic health records	□ Yes □ No	□ High □ Low	
Integrates with other systems (ultrasound, monitors, etc.)	□ Yes □ No	□ High □ Low	
Fits within budget	□ Yes □ No	□ High □ Low	
