# Supplementary material for: Implementing Telemedicine for Neonatal Care: Tutorial on a Practical Toolkit Based on Multinational Experience
Source: J Med Internet Res. 2026 Sep 30;28:e90592. doi: 10.2196/90592 (PMC13626644; doi:10.2196/90592)
Supplement: Supplementary file 5 [file jmir-v28-e90592-s004.doc]

**Evaluation Methods**

| **Evaluation Methods** | **Example** | **Details** | **References** |
| --- | --- | --- | --- |
| **Cognitive Load** | *1. NASA task load* | - mental demand - physical demand - temporal demand - performance - effort - frustration | [1] |
| **User experience** | *2. Telehealth Usability Questionnaire (TUQ)* | - usefulness - rase of use and Learnability - interface quality - interaction quality | [2] |
|  | *3. Telemedicine Satisfaction Questionnaire (TSQ)* | - patient satisfaction - quality of care provided | [3] |
| **Implementation process** | *4. RE-AIM Evaluation Tool* | - reach - effectiveness - adoption of Intervention | [4] |
| **Clinical impact** | *5. IAM (= Information Assessment Method) Evaluation Tool* | - background - usage - purpose - functions | [5] |
| **Usage and benefit of telemedicine** | *6. MAST Evaluation for Telemedicine* | - patient and the technology - patient safety - clinical effectiveness - patient perspectives - economic aspects - organizational aspects - legal and ethical aspects | [6] |
| **Implementation Outcome Repository** | *7. Implementation Outcome Repository* | - implementation outcomes - quantitative measurement instruments - acceptability - feasibility | [7] |

References

1.	Development of NASA-TLX (Task Load Index): Results of Empirical and Theoretical Research. In: Advances in Psychology [Internet]. Elsevier; 1988 [cited 2025 Dec 1]. p. 139–83. Available from: <https://linkinghub.elsevier.com/retrieve/pii/S0166411508623869>

2.	Parmanto B, Lewis, Jr. AN, Graham KM, Bertolet MH. Development of the Telehealth Usability Questionnaire (TUQ). Int J Telerehabilitation. 2016 July 1;8(1):3–10.

3.	Yip MP, Chang AM, Chan J, MacKenzie AE. Development of the Telemedicine Satisfaction Questionnaire to evaluate patient satisfaction with telemedicine: a preliminary study. J Telemed Telecare. 2003 Feb 1;9(1):46–50.

4.	Glasgow RE, Vogt TM, Boles SM. Evaluating the public health impact of health promotion interventions: the RE‑AIM framework. *Am J Public Health*. 1999;89(9):1322‑7.

5.	Bindiganavile Sridhar S, Pluye P, Grad R. In pursuit of a valid information assessment method for continuing education: a mixed methods study. BMC Med Educ. 2013 Dec;13(1):137.

6.	Kidholm K, Clemensen J, Caffery LJ, Smith AC. The Model for Assessment of Telemedicine (MAST): A scoping review of empirical studies. J Telemed Telecare. 2017 Oct;23(9):803–13.

7.	Implementation Outcome Repository. (n.d.). *Home*. Retrieved December 1, 2025, from <https://implementationoutcomerepository.org/>
